# Supplementary material for: Mechanism of beta-arrestin 1 mediated Src activation via Src SH3 domain revealed by cryo-electron microscopy
Source: bioRxiv. 2025 Sep 27:2024.07.31.605623. Preprint. [Version 3] doi: 10.1101/2024.07.31.605623 (PMC11312540; doi:10.1101/2024.07.31.605623)
Supplement: Supplement 1 [file NIHPP2024.07.31.605623v3-supplement-1.pdf]

bioRxiv preprint doi: https://doi.org/10.1101/2024.07.31.605623; this version posted September 27, 2025. The copyright holder for this preprint (which was not certified by peer review) is the author/funder, who has granted bioRxiv a license to display the preprint in perpetuity. It is made available under aCC-BY 4.0 International license.

Supplementary Information

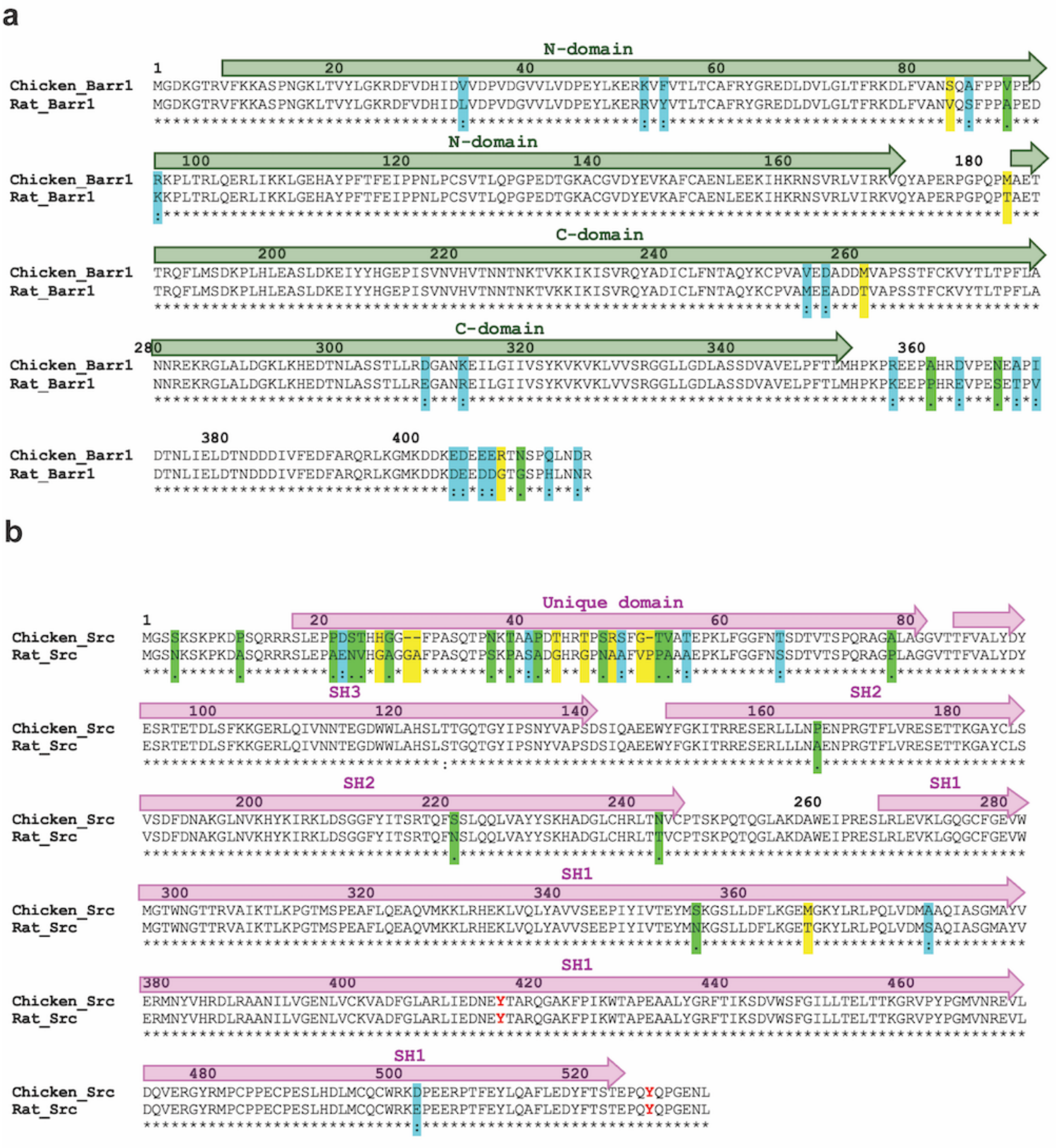

Supplementary Fig. 1. | Alignment of rat (*Rattus norvegicus*) and chicken (*Gallus gallus*) sequences of βarr1 (a) and Src (b). Fully conserved residues are marked with asterisk (*); strongly conserved residues are marked with colon (:) and shaded in cyan; weakly conserved residues are marked with period (.) and shaded in green; non-conserved residues are shaded in yellow. Src active loop tyrosine (Y416) and C-tail tyrosine (Y527) are colored in red. Domains are indicated with arrows and labeled. Sequence alignment was performed in Clustal Omega (https://www.ebi.ac.uk/jdispatcher/msa/clustalo).

bioRxiv preprint doi: https://doi.org/10.1101/2024.07.31.605623; this version posted September 27, 2025. The copyright holder for this preprint (which was not certified by peer review) is the author/funder, who has granted bioRxiv a license to display the preprint in perpetuity. It is made available under aCC-BY 4.0 International license.

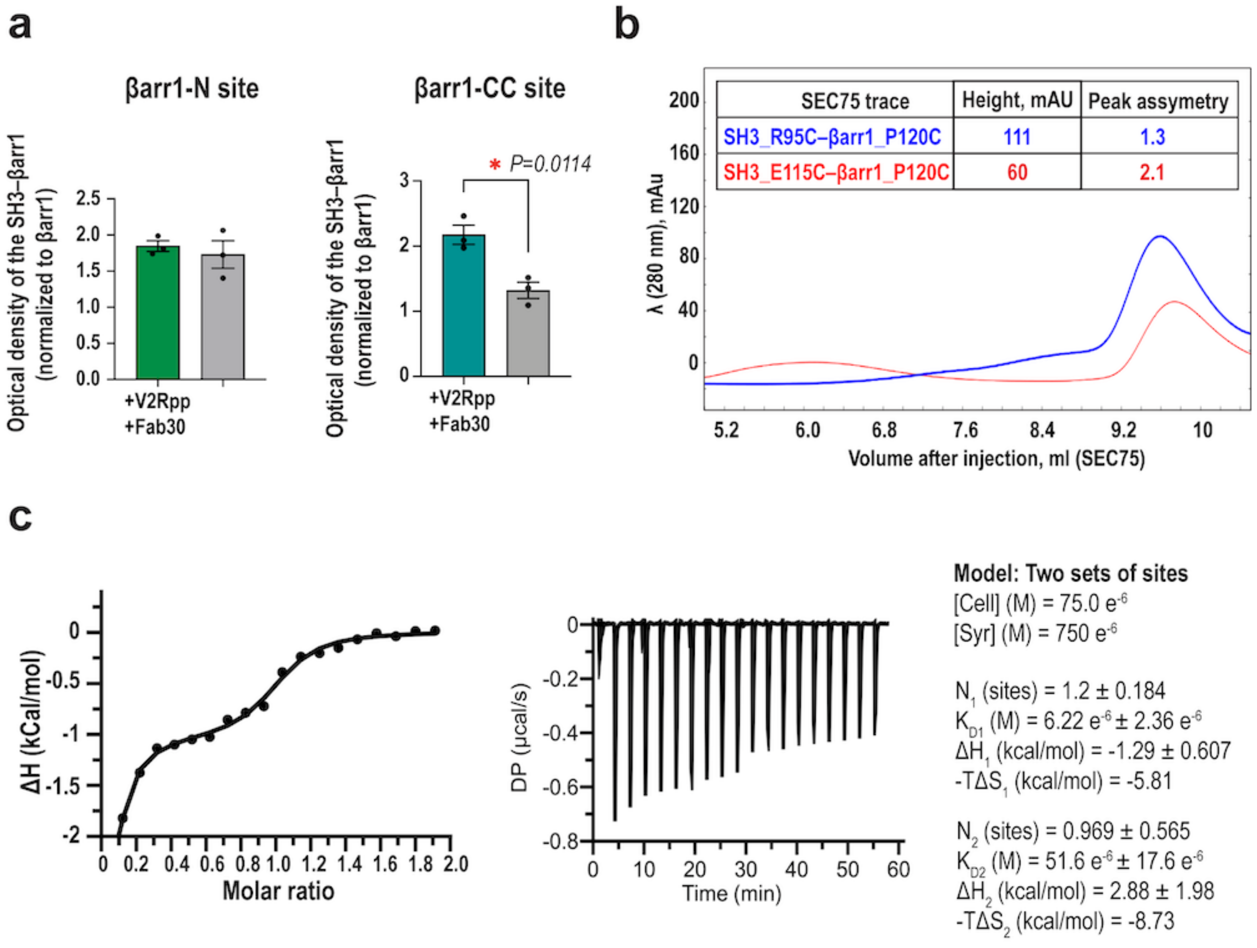

Supplementary Fig. 2. | βarr1 uses two distinct sites to bind SH3. **a,** Densitometry analysis of SH3–βarr1 disulfide trapping with and without V2Rpp (synthetic phosphopeptide mimicking the C-tail of vasopressin 2 receptor) and stabilizing antibody Fab30 (mean ± standard error of mean (SEM), n=3). Statistical analysis was performed using Student's t-test. **b,** Analytical size-exclusion chromatography profile of SH3_R95C–βarr1_P120C and SH3_E115C–βarr1_P120C disulfide trapped complexes. **c,** Isothermal titration calorimetry of SH3 binding to βarr1–V2Rpp. Top panel: integrated heat (after deducting heat of dilution) per injection of SH3 into βarr1–V2Rpp solution in the cell based on the molar ratio of each injection. Bottom panel: raw titration data showing the heat rate associated with each dilution per injection versus time into βarr1–V2Rpp solution in the cell. Right panel: thermodynamic parameters of SH3–βarr1–V2Rpp. The data are representative of three independent experiments.

bioRxiv preprint doi: https://doi.org/10.1101/2024.07.31.605623; this version posted September 27, 2025. The copyright holder for this preprint (which was not certified by peer review) is the author/funder, who has granted bioRxiv a license to display the preprint in perpetuity. It is made available under aCC-BY 4.0 International license.

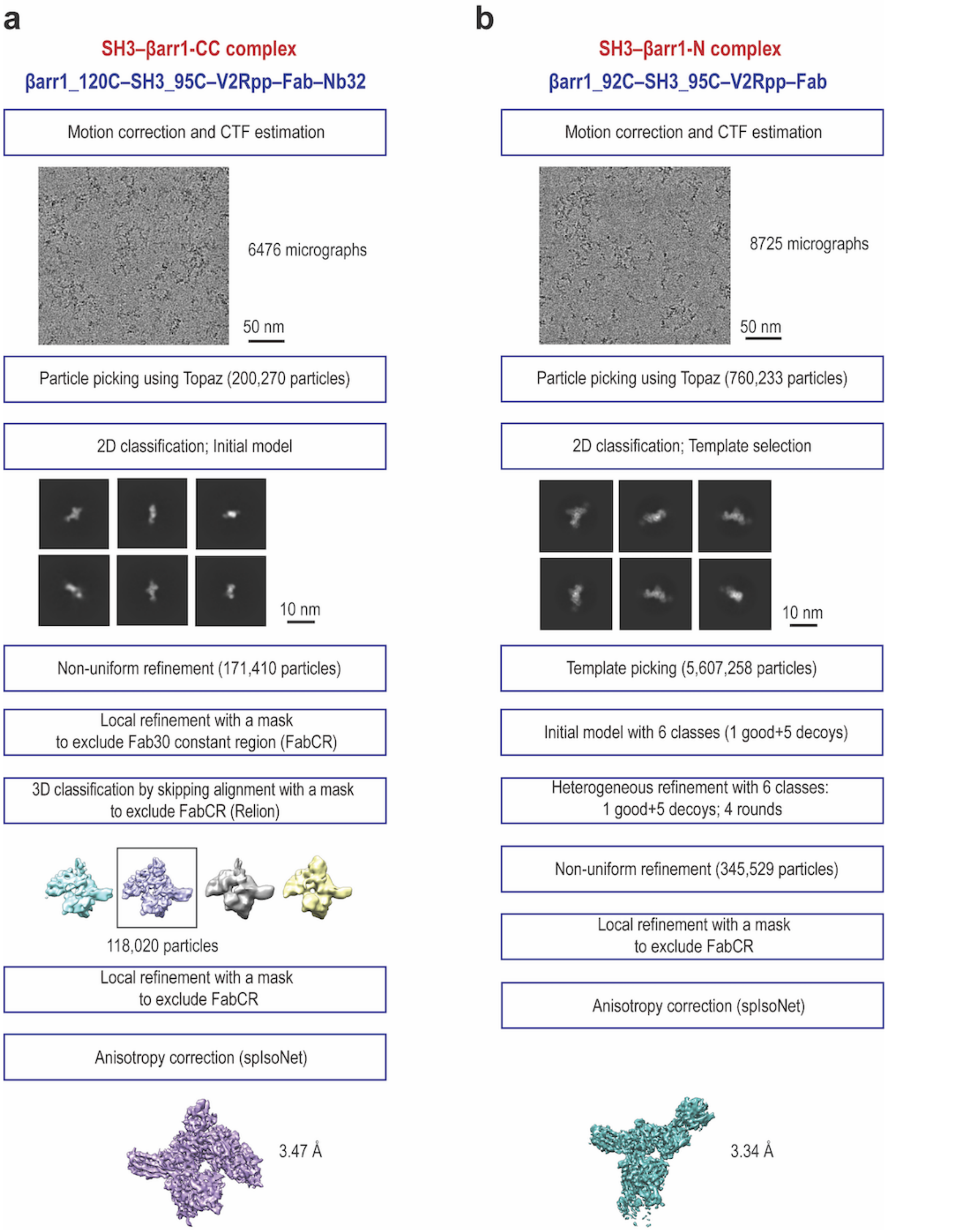

Supplementary Fig. 3. | Flow chart of cryo-EM data processing of SH3–βarr1-CC (a) and SH3–βarr1-N (b) complexes.

bioRxiv preprint doi: https://doi.org/10.1101/2024.07.31.605623; this version posted September 27, 2025. The copyright holder for this preprint (which was not certified by peer review) is the author/funder, who has granted bioRxiv a license to display the preprint in perpetuity. It is made available under aCC-BY 4.0 International license.

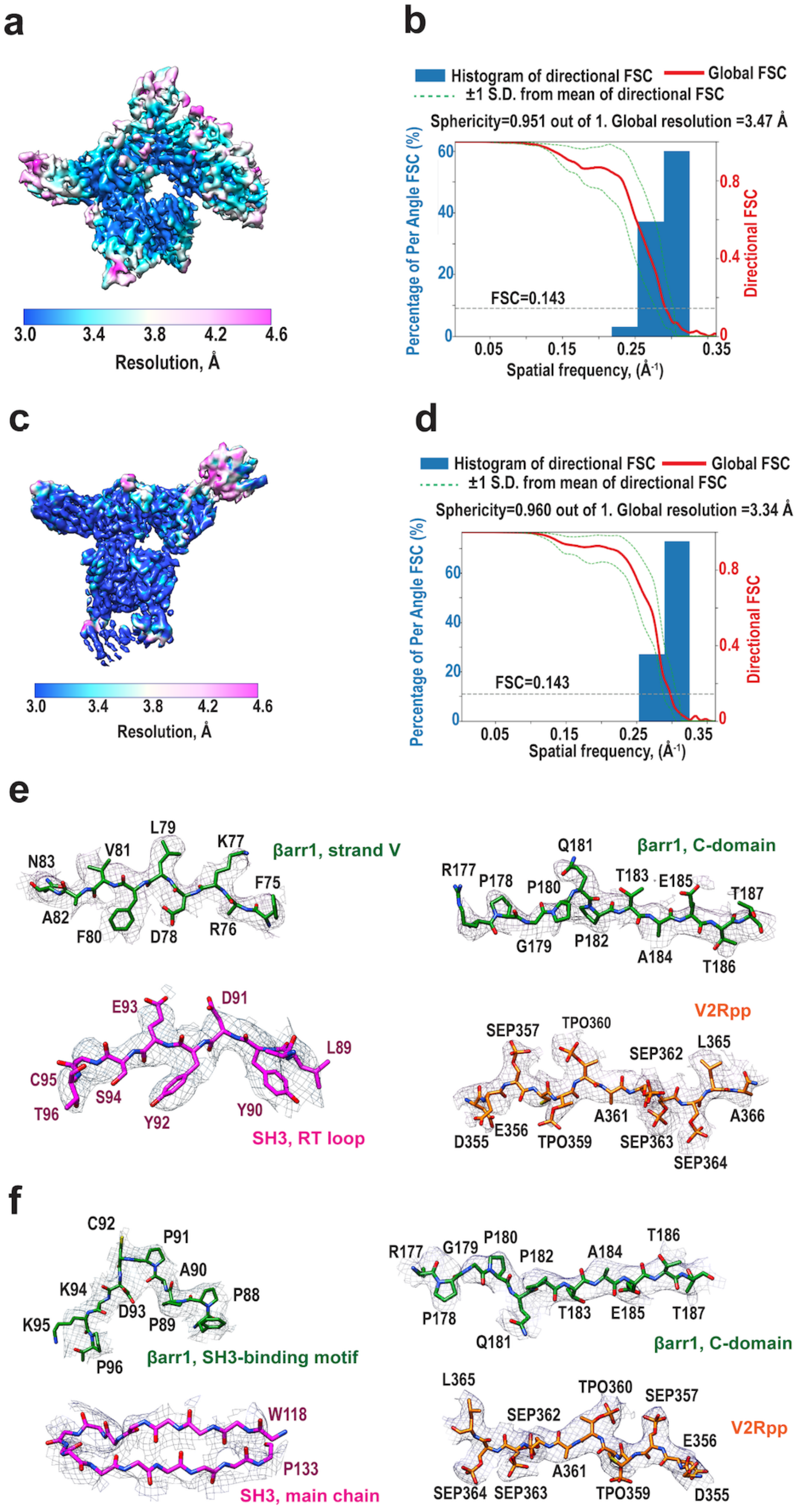

bioRxiv preprint doi: https://doi.org/10.1101/2024.07.31.605623; this version posted September 27, 2025. The copyright holder for this preprint (which was not certified by peer review) is the author/funder, who has granted bioRxiv a license to display the preprint in perpetuity. It is made available under aCC-BY 4.0 International license.

Supplementary Fig. 4. | Cryo-electron microscopy of SH3–βarr1 complexes. a-c, Cryo-EM maps of SH3–βarr1-CC (**a**) and SH3–βarr1-N (**c**) coloured by local resolution; resolution is reported at the Fourier Shell Correlation (FSC) threshold of 0.143. Map contour level is 0.83 for SH3–βarr1-CC (**a**) and 0.50 for SH3–βarr1-N (**c**). **b-d,** Histogram and directional FSC plot measuring directional resolution anisotropy for SH3–βarr1-CC (**b**) and 0.50 for SH3–βarr1-N (**d**). Sphericity values were determined at an FSC threshold of 0.5 with the 3DFSC software.[73] **e-f,** Cryo-EM density at different parts of the SH3–βarr1-CC (**e**) and SH3–βarr1-N (**f**) complexes. The upsample maps are used (0.69 Å/pix); map contour level is 0.3 (SH3–βarr1-CC) and 0.2 (SH3–βarr1-N).

bioRxiv preprint doi: https://doi.org/10.1101/2024.07.31.605623; this version posted September 27, 2025. The copyright holder for this preprint (which was not certified by peer review) is the author/funder, who has granted bioRxiv a license to display the preprint in perpetuity. It is made available under aCC-BY 4.0 International license.

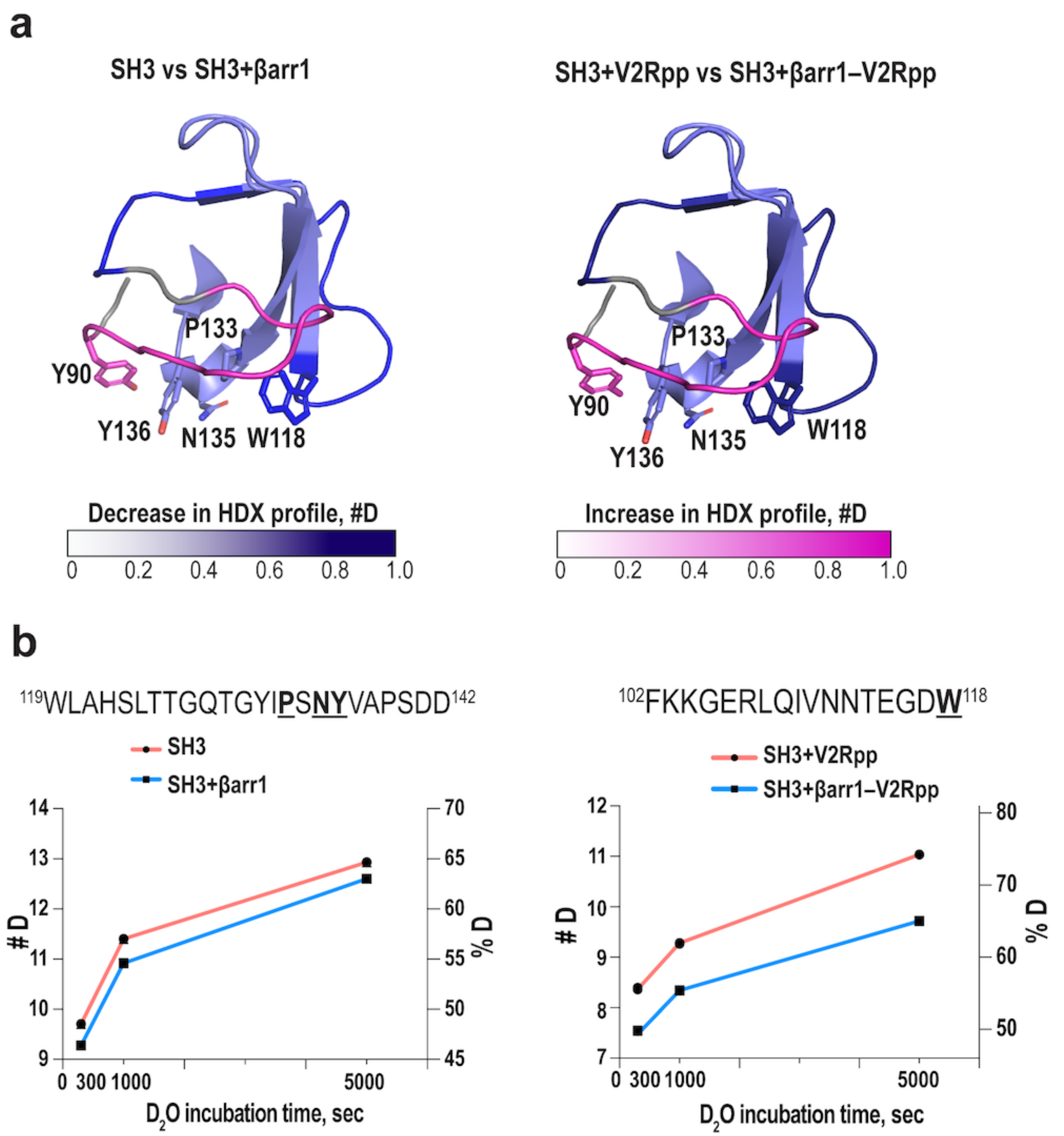

Supplementary Fig. 5. | HDX profile changes in SH3 upon co-incubation with βarr1. a, Structure of SH3 (PDB: 2PTK) upon co-incubation with free βarr1 (left panel) or V2Rpp-activated βarr1 (right panel). Regions with decreased and increased deuterium uptake are shaded in dark-blue and magenta, respectively. Only regions that showed differences between the states in deuterium uptake (>0.2 #D) in at least two overlapping peptides are indicated. **b,** The HDX profile of representative peptides in SH3. The residues interacting with βarr1 in the structures are shown in bold and underlined.

bioRxiv preprint doi: https://doi.org/10.1101/2024.07.31.605623; this version posted September 27, 2025. The copyright holder for this preprint (which was not certified by peer review) is the author/funder, who has granted bioRxiv a license to display the preprint in perpetuity. It is made available under aCC-BY 4.0 International license.

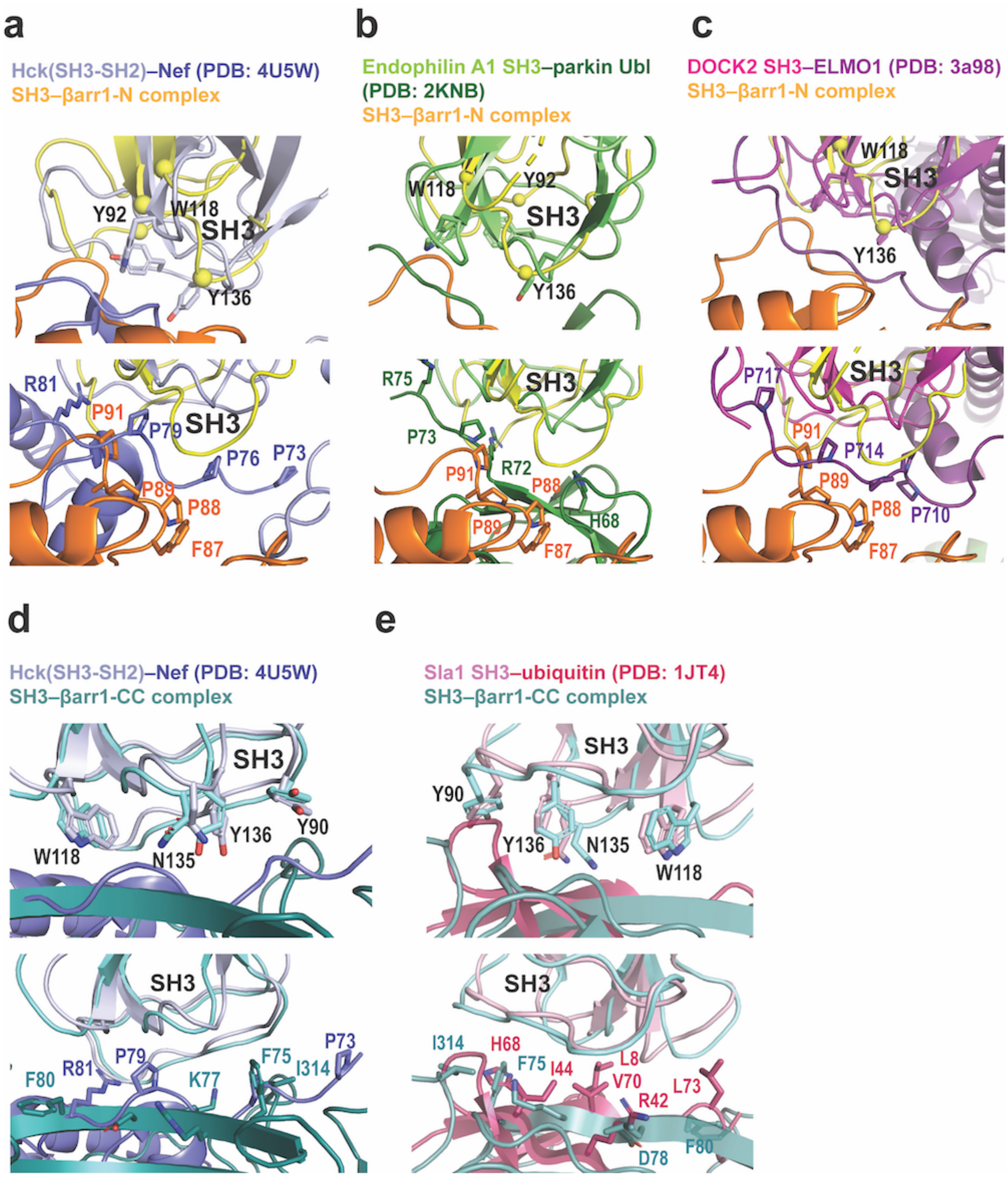

Supplementary Fig. 6. | Structural superpositions of SH3 domains in SH3–βarr1-N (yellow), SH3–βarr1-CC (cyan) and other SH3-binding complexes. a, SH3–βarr1-N (yellow) and Hck(SH3-SH2)-Nef (slate). **b,** SH3–βarr1-N (yellow) and endophilin A1 SH3–parkin Ubl (green). **c,** SH3–βarr1-N (yellow) and DOCK2 SH3–ELMO 1 (magenta). **d,** SH3–βarr1-CC (cyan) and Hck(SH3-SH2)-Nef (slate). **e,** SH3–βarr1-CC (cyan) and Sla1 SH3-ubiquitin (pink). The top panel shows the interacting residues in SH3 (Src SH3 numbering is used), the bottom panel shows interacting residues in βarr1 and the SH3-binding protein.

bioRxiv preprint doi: https://doi.org/10.1101/2024.07.31.605623; this version posted September 27, 2025. The copyright holder for this preprint (which was not certified by peer review) is the author/funder, who has granted bioRxiv a license to display the preprint in perpetuity. It is made available under aCC-BY 4.0 International license.

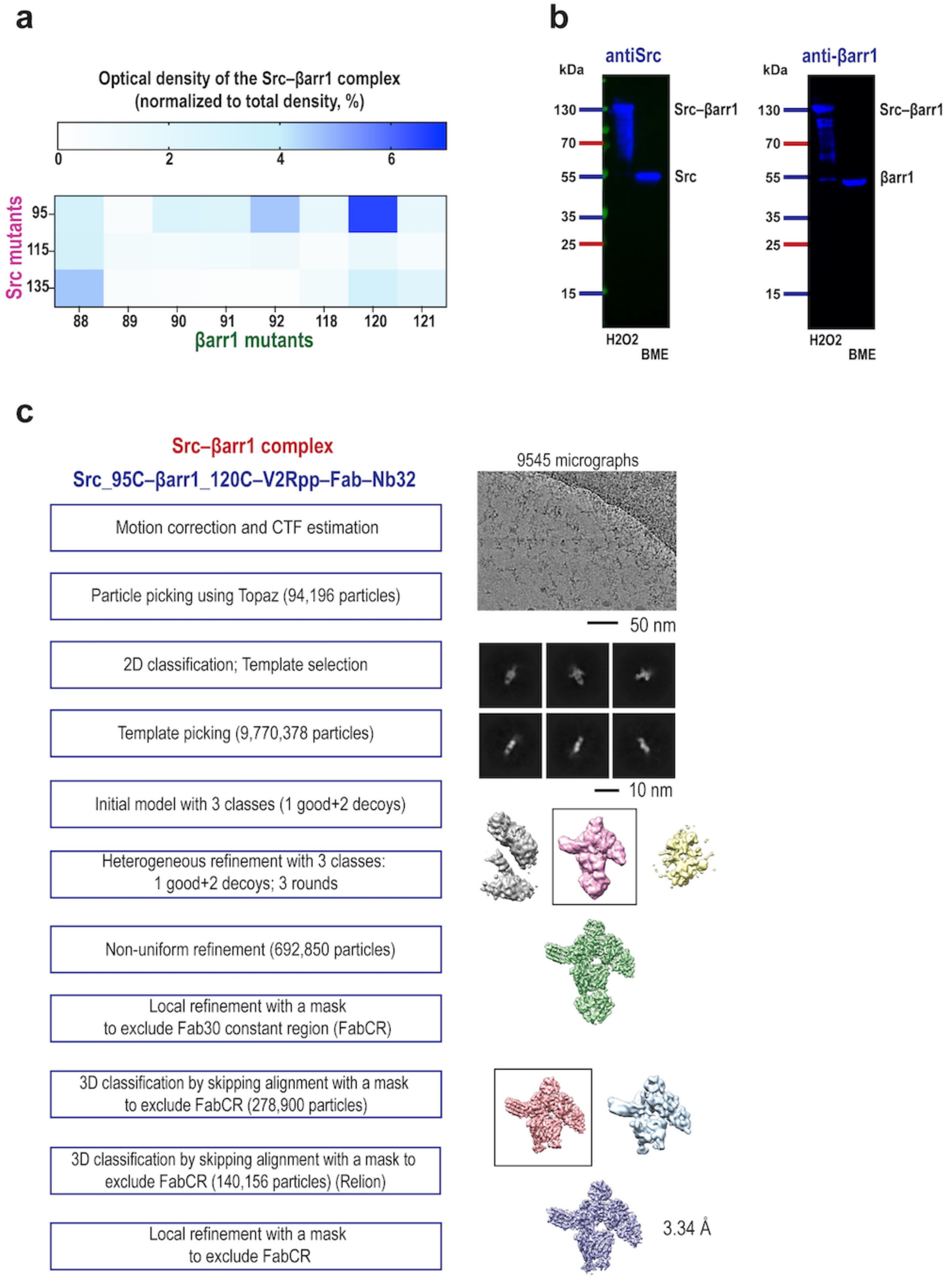

bioRxiv preprint doi: https://doi.org/10.1101/2024.07.31.605623; this version posted September 27, 2025. The copyright holder for this preprint (which was not certified by peer review) is the author/funder, who has granted bioRxiv a license to display the preprint in perpetuity. It is made available under aCC-BY 4.0 International license.

Supplementary Fig. 7. | Complex formation and cryo-EM data processing of Src–βarr1-CC. **a,** Complex formation between Src and βarr1 mutants revealed by disulfide trapping; densitometry analysis of Coomassie blue gels. The Src–βarr1-CC complex band was normalized to the total density of all bands in each sample (mean, n=3-9). Prior to disulfide trapping reactions, βarr1 was activated by V2Rpp. **b,** Western blot of disulfide trapping reaction with βarr1_120C and Src _R95C. 130-kDa band is detected by both anti-βarr1 (A1CT) and anti-Src antibodies, confirming that the band is the covalent Src–βarr1-CC complex. **c,** Flow chart of cryo-EM data processing of the Src–βarr1-CC complex.

bioRxiv preprint doi: https://doi.org/10.1101/2024.07.31.605623; this version posted September 27, 2025. The copyright holder for this preprint (which was not certified by peer review) is the author/funder, who has granted bioRxiv a license to display the preprint in perpetuity. It is made available under aCC-BY 4.0 International license.

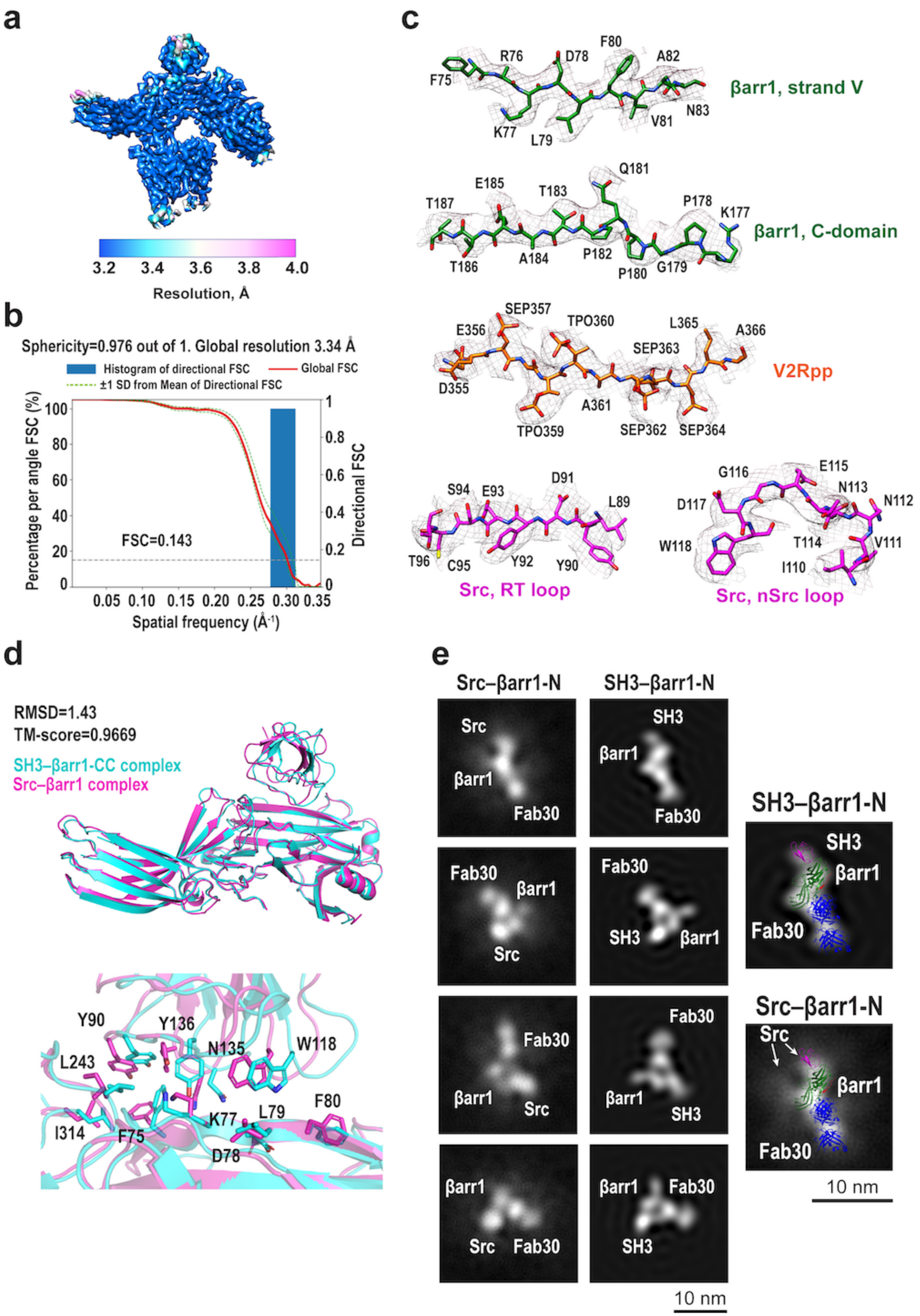

bioRxiv preprint doi: https://doi.org/10.1101/2024.07.31.605623; this version posted September 27, 2025. The copyright holder for this preprint (which was not certified by peer review) is the author/funder, who has granted bioRxiv a license to display the preprint in perpetuity. It is made available under aCC-BY 4.0 International license.

Supplementary Fig. 8. | Cryo-electron microscopy of Src–βarr1 complexes. a, Cryo-EM map of the Src–βarr1-CC complex coloured by local resolution; resolution is reported at the FSC threshold of 0.143. Map contour level is 0.55. **b,** Histogram and directional FSC plot. A sphericity of 0.976 determined at an FSC threshold of 0.5 indicates isotropic angular distribution. Directional FSC determination was performed with the 3DFSC software.[73] **c,** Cryo-EM density at different parts of Src–βarr1-CC map. The upsample map is used (0.72 Å/pix); map contour level is 0.1. **d,** The structural superposition of the Src–βarr1-CC and SH3–βarr1-CC complexes (Fab30, V2Rpp and Nb32 are not shown). RMSD and the TM-score of the structures was calculated using the TM-score function[74]. **e,** Matching of the 2D classes of the Src–βarr1-N complex with the projections of the SH3–βarr1-N map low-pass filtered to 25 Å using cluster selection mode in Reference Based Auto Select 2D module in *CryoSPARC* (left panel) and representative 2D classes of the complexes with the fitted model of SH3–βarr1-N (right panel).

bioRxiv preprint doi: https://doi.org/10.1101/2024.07.31.605623; this version posted September 27, 2025. The copyright holder for this preprint (which was not certified by peer review) is the author/funder, who has granted bioRxiv a license to display the preprint in perpetuity. It is made available under aCC-BY 4.0 International license.

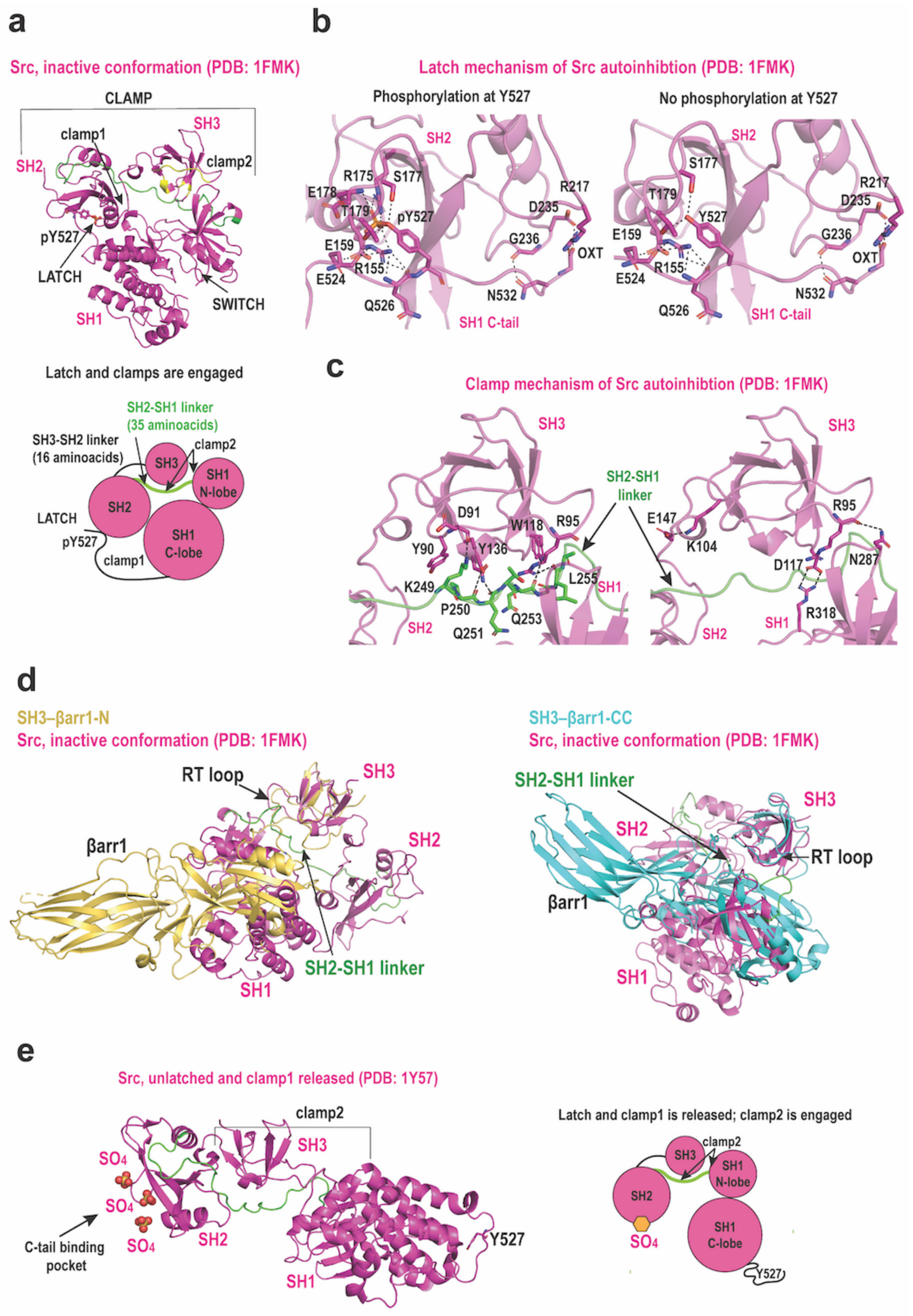

bioRxiv preprint doi: https://doi.org/10.1101/2024.07.31.605623; this version posted September 27, 2025. The copyright holder for this preprint (which was not certified by peer review) is the author/funder, who has granted bioRxiv a license to display the preprint in perpetuity. It is made available under aCC-BY 4.0 International license.

Supplementary Fig. 9. | Autoinhibition of Src. a, Structure of autoinhibited Src (PDB: 1FMK), magenta. Parts of SH3 interacting with βarr1 are colored in yellow. SH2-SH1 linker is colored in green; phosphorylated Y527 is labeled. **b-c,** Latch (**b**) and clamp (**c**) mechanisms of Src autoinhibition (PDB: 1FMK), cartoon representation (magenta). The hydrogen bonds are depicted as dashed lines; interacting residues are labeled. **d,** Structural superpositions of SH3–βarr1-N (yellow) and SH3–βarr1-CC (cyan) with inactive Src (PDB: 1FMK, magenta). **e,** Structure of unphosphorylated Src (PDB: 1Y57), magenta. SH2-SH1 linker is colored in green; phosphorylated Y527 is labeled. Note that the C-tail binding pocket on SH2 is occupied by sulfate ions originating from crystallization solution.

bioRxiv preprint doi: https://doi.org/10.1101/2024.07.31.605623; this version posted September 27, 2025. The copyright holder for this preprint (which was not certified by peer review) is the author/funder, who has granted bioRxiv a license to display the preprint in perpetuity. It is made available under aCC-BY 4.0 International license.

Supplementary Table 1. Cryo-EM data collection, refinement and validation statistics

	**SH3_95C–βarr1_120C–V2Rpp–Fab30–Nb32 (SH3–βarr1-CC)** (EMD-45977) (PDB 9CX3)	**SH3_95C–βarr1_92C–V2Rpp–Fab30 (SH3–βarr1-N)** (EMD-45982) (PDB 9CX9)	**Src_95C–βarr1_120C–V2Rpp–Fab30–Nb32 (Src–βarr1-CC)** (EMD-44881) (PDB 9BT8)
**Data collection and processing**			
Magnification	81,000	81,000	81,000
Voltage (kV)	300	300	300
Electron exposure (e–/Å^2)	58.5	53.8	54.6
Defocus range (μm)	-0.8 to -2.5	-0.8 to -2.5	-0.8 to -2.5
Pixel size (Å)	1.08 (collection)<br>1.3824 (final)	1.08 (collection)<br>1.3824 (final)	1.08 (collection)<br>1.44 (final)
Symmetry imposed	C1	C1	C1
Initial particle projections (no.)	200,270	5,607,258	9,770,378
Final particle projections (no.)	118,020	345,529	140,156
Map resolution (Å)	3.47	3.34	3.32
FSC threshold	0.143	0.143	0.143
Map resolution range (Å)	3.10-6.97	3.05-6.90	3.23-11.33
**Refinement**			
Initial model used (PDB code)	6NI2	4JQI	8U7A
Model resolution (Å)	3.47	3.3	3.2
FSC threshold	0.143	0.143	0.143
Map sharpening *B* factor (Å^2)	-80.3	-83.2	-77.5
Model composition			
Non-hydrogen	5627	4580	5713
atoms	720	609	728
Protein residues	0	0	0
Ligands			
B factors (Å^2)			
Protein	20.22/103.43/47.54	8.88/113.35/37.29	10.51/115.11/53.64
(min/max/mean)	---	---	
Ligand			
R.m.s. deviations			
Bond lengths (Å)	0.005	0.004	0.004
Bond angles (°)	0.709	0.988	0.572
Validation			
MolProbity score	1.93	1.56	1.55
Clashscore	11.22	7.59	6.88
Poor rotamers (%)	0.16	0.21	0.32
Ramachandran plot			
Favored (%)	94.78	97.25	97.00
Allowed (%)	5.22	2.75	3.00
Disallowed (%)	0.00	0.00	0.00
